# Strengthening rehabilitation to reduce burn contractures and disability in sub Saharan Africa

**DOI:** 10.3389/fresc.2026.1687061

**Published:** 2026-04-28

**Authors:** Shani Kondo Omari, Lydia Issaria Mosses

**Affiliations:** Research, Policy and Innovation Department, Tanzania Inclusion and Development Forum (TIDF), Dar es Salaam, Tanzania

**Keywords:** global health equity, burn contractures, disability, disability inclusion, disability prevention, functional recovery, health systems strengthening, rehabilitation

## Abstract

Burn injuries remain a major yet under-recognized driver of long-term disability in low-and middle-income countries particularly Sub-Saharan Africa. While survival rates have improved in some settings, recovery often ends at hospital discharge. Thousands of survivors particularly children, women, and those living in poverty develop preventable burn contractures due to delayed or absent rehabilitation. These disabling complications restrict movement, limit education and employment opportunities, and reinforce cycles of poverty and exclusion. It has been shown that early, low-cost rehabilitation including positioning, splinting, and range-of-motion exercises initiated within days of injury can significantly reduce contracture formation and improve functional outcomes. Yet rehabilitation remains fragmented, underfunded, and largely excluded from national burn management guidelines and Universal Health Coverage (UHC) benefit packages across many Sub-Saharan African countries. This article argues that burn rehabilitation must be repositioned from an optional add-on to a core component of comprehensive burn care and a pillar of disability-inclusive health systems. It examines systemic barriers including workforce shortages, policy invisibility, infrastructure gaps, financial risk protection failures, and sociocultural stigma. It further highlights practical, scalable solutions such as early bedside rehabilitation protocols, task-shifting models, community-based rehabilitation, survivor-led peer support, and integration into national health financing frameworks. Strengthening rehabilitation is not a competing priority it completes burn care. By embedding functional recovery into policy and practice, Sub-Saharan Africa can move beyond survival toward dignity, inclusion, and long-term productivity for burn survivors.

## 1 Background

Burn injury is defined as damage to the skin or underlying tissues caused by heat, electricity, chemicals, or radiation. It is more than a physical wound; it is a trauma that affects the entire trajectory of a person's life. According to the World Health Organization (WHO), an estimated 180,000 deaths annually are caused by burns, with the vast majority occurring in low- and middle-income countries (LMICs), especially in Sub-Saharan Africa and South Asia (1). But the true burden of burns lies not only in mortality but also in the lives forever changed by disability (1). Disability, according to the World Health Organization (WHO), is not merely a health problem, but rather a complex interaction between an individual's health condition and contextual factors, including environmental and personal factors. It refers to difficulties in functioning at the body, person, or societal level due to impairments, activity limitations, and participation restrictions. In simpler terms, disability arises when there is a mismatch between a person's abilities and the physical, social, or attitudinal environment in which they live (2). One of the most disabling outcomes of burn injuries is the development of burn contractures, which is a pathological scarring that leads to the tightening of skin, muscles, and tendons. Contractures severely restrict movement of joints and are often disfiguring, especially when they affect the neck, hands, or face (3).

In Sub-Saharan Africa, where children often play near open fires, families cook over unsafe stoves, and access to emergency care is delayed, burns are a daily reality (1). In many settings, survivors particularly those who are poor, young, or female face a high risk of contractures due to delayed treatment, limited access to surgical correction, and critically, inadequate rehabilitation services (4). Too often, survival comes at the cost of permanent functional impairment. The consequences extend beyond the clinical outcome. Once wounds have healed, many children are unable to return to school, caregivers carry lifelong physical and economic burdens, and communities quietly absorb the cost of lost productivity and restricted participation outcomes that could have been mitigated through timely rehabilitation. While acute burn care such as debridement, fluid resuscitation, and infection control has advanced in several parts of Sub Saharan Africa regions, rehabilitation remains the most neglected component (4, 5). Yet, evidence shows that early, low-cost rehabilitation (including splinting, positioning, and physical therapy) can prevent or greatly reduce the severity of contractures (6–8). The injustice lies not in the injury itself, but in the structural failure to provide continuity of care, to move beyond simply helping someone survive, toward helping them live well.

These systemic gaps expose the urgent need for disability-inclusive health systems in which rehabilitation is embedded as an essential health service within UHC frameworks, rather than treated as a secondary or specialized add-on. Burn rehabilitation must therefore be reframed from an optional medical “afterthought” into a core component of Universal Health Coverage (UHC) and the fundamental right to health. A disability-inclusive health system is defined by its alignment with the United Nations Convention on the Rights of Persons with Disabilities (CRPD), which underscores that access to rehabilitation is a fundamental human right essential for preventing avoidable disability and enabling survivors to reach the highest possible standard of health (9). In disability – inclusive health system, rehabilitation is not treated as an optional medical “afterthought” or a secondary add-on, but is instead embedded as an essential health service within national Universal Health Coverage (UHC) benefit packages and clinical protocols. By embedding rehabilitation as an essential service within UHC frameworks, health systems can move beyond survival to ensure functional recovery and social reintegration (9).

This model necessitates a multisector commitment to ensuring that the care trajectory for burn survivors extends beyond acute surgical survival to include longitudinal functional recovery, psychosocial support, and community reintegration, specifically targeting the structural and financial barriers that often force marginalized populations into cycles of permanent impairment and poverty (10). Importantly, engaging burn survivors and their families in the design and evaluation of rehabilitation programs strengthens contextual relevance, improves adherence to therapy, and helps address inequities in access particularly for women, children, and marginalized populations. Centering lived experience is not only good practice; it is fundamental to building equitable, responsive systems of care (11).

Critical to this inclusive model is the integration of rehabilitation into Universal Health Coverage (UHC). Rather than an optional “afterthought,” rehabilitation must be defined as an “essential health service' embedded within national clinical protocols and Essential Health Benefit Packages (EHBP). By reframing burn recovery through this rights-based and financing lens, health systems move beyond acute surgical survival toward a standard of functional recovery and social dignity.

## 2 From survival to disability: the unfinished story of burn recovery

In many burn centers across Sub-Saharan Africa, hospital discharge often marks the end of care rather than the beginning of recovery, as follow-up and structured rehabilitation services after discharge are rarely available, leaving many survivors without ongoing support for functional recovery (6, 10, 12–14). This gap in continuity of care often described as the “discharge gap” represents a systemic failure where survivors return home with healing wounds, restricted movement, and no structured plan for how to regain mobility, independence, or social inclusion. Survivors frequently return home with healing wounds, yet without longitudinal follow-up, rehabilitation is not sustained beyond the acute phase. This transition from acute ward survival to long-term community reintegration represents the most critical phase for a survivor's livelihood, as it determines whether a healed wound results in a return to productivity or a descent into permanent disability.

Despite international recommendations established by the International Society for Burn Injuries (ISBI) and the integrated quality improvement frameworks championed by Interburns, rehabilitation services remain poorly integrated into acute burn care, particularly in resource-constrained settings (10, 12, 15, 16). Strengthening this neglected pillar requires integrating rehabilitation into Disability-Inclusive Health Systems, ensuring that longitudinal follow-up is not a luxury but a standard clinical protocol. This discharge gap is further worsened by limited financial risk protection, which forces disproportionately poor survivors to abandon therapy. Integrating rehabilitation into essential health benefit packages is therefore crucial for achieving disability-inclusive health systems. Without these protections, contractures and long-term impairments are not only clinical complications but also stark indicators of systemic inequities in health care access and coverage.

Burn contractures are not rare complications; they are predictable outcomes in the absence of early, consistent rehabilitation. The primary biological driver of this disability occurs during the wound-healing phase: when joints are not mobilized, collagen fibers are laid down in disorganized, shortened patterns rather than following a functional alignment. This aberrant maturation results in thick, inelastic fibrotic tissue that pulls the body into painful, fixed deformities, especially around the neck, axilla, elbows, and hands (17). According to studies up to 35% of moderate-to-severe burn survivors develop function-limiting contractures, particularly when rehabilitation is delayed or omitted (18). These are not isolated cases, rather they are the outcomes of systemic failures to intervene during the critical window of scar maturation especially in settings where rehabilitation is seen as non-essential (19, 20).

The consequences are lifelong. A child may lose the ability to write, eat independently, or dress themselves. A woman may be unable to breastfeed or care for her family due to contractures in her upper limbs. A manual laborer may lose their livelihood due to impaired grip or mobility. These consequences extend beyond the physical; the psychosocial toll is equally profound. Burn survivors often experience social isolation, stigma, and depression, especially when visible scarring affects the face or hands, which can negatively impact their social participation and quality of life (21). Girls and women, in particular, may experience early school dropout, marriage challenges, or abandonment, reinforcing cycles of poverty and marginalization.

Yet this suffering is largely preventable. Studies have shown that early interventions within 5–7 days post injury utilizing basic range-of-motion exercises and positioning, can dramatically reduce the risk of severe contractures (6, 7). A trial conducted in China has demonstrated that even task-shifted rehabilitation, led by nurses, significantly improves joint mobility and quality of life “post-burn” (22). Survivors do not need to live in pain or silence. But in many systems, they are forgotten the moment the wound closes. Bridging this gap requires not only clinical solutions but also policy commitment, resource allocation, and cultural change (23).

## 3 Barriers to integration of rehabilitation in burn care

Despite well-documented evidence supporting burn rehabilitation, its implementation across many low-resource settings remains fragmented, underfunded, and inconsistently delivered (24, 25). The absence of rehabilitation is not simply a resource issue; it reflects broader structural and systemic challenges within health systems. These barriers, collectively, create a systemic blind spot, where the health system achieves survival, but not recovery.

### 3.1 Workforce shortages critical need for task-shifting

A critical barrier is the scarcity of trained rehabilitation personnel. According to WHO's *Rehabilitation in Health Systems* report, over half the population in LMICs lack access to rehabilitation services, largely due to workforce gaps (24). In Sub-Saharan Africa, some countries have fewer than 0.5 physiotherapists per 10,000 people, and occupational therapy is virtually absent from public health systems (26).

In many district and regional hospitals, no rehabilitation professionals are present (27). Surgical or nursing teams with limited or no training in “post-burn” functional recovery typically manage burn care. This workforce gap underscores the urgent need to expand task-shifting models to train frontline workers and invest in formal academic degree programs to build local specialist capacity. Without task-shifting or formal training programs, rehabilitation becomes dependent on NGOs or urban centers (28, 29).

### 3.2 Policy invisibility and the lack of financial risk protection

Despite the heavy burden of burn-related disability across Sub-Saharan Africa. National health strategies across the region frequently neglect rehabilitation. A review found that none of the 48 countries included specific policies or budget allocations for post-acute burn rehabilitation, even where acute care was mentioned (26, 30, 31). This omission contributes to ongoing underfunding of training programs, rehabilitation infrastructure, and assistive tools. Without inclusion in national management guidelines or Universal Health Coverage (UHC) benefit packages, rehabilitation remains an “optional” luxury. This policy void forces survivors to abandon therapy due to cost, leading to a significant reintegration burden where survivors with contractures have a 55% lower probability of returning to work compared to those without (26).

### 3.3 Fragmented and incomplete care pathways and discharge gap

Burn rehabilitation is often seen as a separate service, rather than a continuum of care. There is a profound disconnect between hospital discharge and community reintegration. In many centers, discharge marks the end of care rather than the beginning of recovery, with no structured follow-up systems. Patients are frequently discharged with healing wounds, may be told to return “if needed,” without a clear timeline or structured follow-ups, allowing the contractures to progress silently and unchecked once the patient has left the clinical environment (32). Addressing this requires the launch of early bedside protocols within 48–72 h and the development of community-based rehabilitation (CBR) arms to bridge the transition from hospital to home.

### 3.4 Infrastructure deficits and the absence of feasible data systems

Basic rehabilitation tools such as splints, pressure garments, and assistive devices are either unavailable or unaffordable. Therapy rooms are rare in district hospitals. In some settings, even improvised tools like banana leaf splints or cardboard pressure masks are used; but they are often applied without guidance, leading to poor outcomes (33). Furthermore, the lack of computerized medical records and standardized definitions for contractures makes it impossible to track recovery outcomes accurately. This lack of evidence-based tracking highlights the necessity for robust data systems that utilize locally meaningful and feasible indicators such as joint function and return-to-school rates to inform resource allocation.

### 3.5 Sociocultural stigma and the underutilization of lived experience

Stigma and cultural beliefs such as the perception that deformity is an “inevitable” outcome or even a result of bewitchment discourage families from seeking therapy. The trauma of “losing face” and social mockery often lead to profound isolation and school dropout, particularly for girls (30). Addressing these deep-seated barriers requires clinical solutions as well as survivor-led peer support and advocacy, which empowers survivors to navigate social mockery. The survivor led groups are necessary to rebuild survivor identity, mitigate social isolation, and demand service equity through shared lived experience (24).

## 4 Recommendations: closing the rehabilitation gap in burn care

To address the systemic neglect of rehabilitation in burn care across Sub-Saharan Africa, targeted, multisector interventions must be urgently pursued. It is essential to acknowledge that the effectiveness of rehabilitation is closely dependent on the quality and availability of acute burn care, as timely wound closure and skin grafting are prerequisites for optimal functional outcomes (10, 29, 34). Burn recovery must be viewed as a multidisciplinary process involving surgeons, nurses, physiotherapists, dietitians, and social workers to address the nutritional, surgical, and psychological factors that influence scar maturation (29, 35). Furthermore, implementing decentralized and robust follow-up systems is vital to bridge the “discharge gap' and support early identification of emerging disabilities as scars continue to evolve long after discharge, especially in children (36). As countries pursue Universal Health Coverage and disability-inclusive development goals, burn rehabilitation must be recognized as an essential health service and not a luxury. The following recommendations provide actionable strategies to ensure burn survivors receive timely, appropriate, and sustained rehabilitation services:

### 4.1 Integrate rehabilitation into national burn management guidelines

Rehabilitation should be formally recognized as an essential component of comprehensive burn care and explicitly incorporated into national clinical guidelines, standardized treatment protocols, and health system performance frameworks. Clear, structured care pathways from acute management through long-term follow-up and community reintegration are necessary to ensure rehabilitation is initiated early, and sustained throughout recovery.

Integrating rehabilitation into national strategies aligns with the priorities of the World Health Organization, Rehabilitation 2030 Call to Action, which urges countries to strengthen rehabilitation services across all levels of care, particularly through early intervention and integration into primary health systems (37). It also reflects the commitments outlined in the United Nations Convention on the Rights of Persons with Disabilities, which emphasizes access to timely and appropriate rehabilitation as a fundamental right (9).

Furthermore, such integration supports the regional mission of the Pan African Burn Society (PABS) to standardize high-quality burn care and prevention across Africa. Recognizing early rehabilitation as a core element of the burn care continuum will not only improve functional outcomes but also promote long-term participation, inclusion, and quality of life for burn survivors.

### 4.2 Launch early rehabilitation protocols in all burn units

Hospitals and health centers managing burns should be required to implement early rehabilitation protocols within the first 48–72 h post-injury. This includes simple bedside interventions such as passive range of motion, optimal positioning, splinting, and patient/caregiver education.

### 4.3 Expand task-shifting models to include burn rehabilitation

Addressing the critical shortage of specialized personnel in Sub-Saharan Africa, where some nations have fewer than 0.5 physiotherapists per 10,000 people, health systems must expand task-shifting models to ensure recovery is not a luxury reserved for urban centers (24). To operationalize task-shifting, national health authorities and academic institutions must integrate basic burn rehabilitation competencies specifically positioning, splinting, and range-of-motion exercises into the formal curricula for nurses and clinical officers (24, 26, 35). These non-specialist providers should be supervised by existing specialized therapists or surgical teams to ensure that early protocols are launched within the first 48–72 h post-injury, bridging the gap in rural areas where specialized personnel are absent (6).

Evidence from trials in Uganda, Bangladesh, and China demonstrates that task-shifted rehabilitation led by nurses or community workers significantly improves joint mobility and quality of life for burn survivors (30, 36, 38). The feasibility of task-shifting is illustrated by the nurse-led transitional burns rehabilitation program (4Cs-TBuRP), which was designed to bridge the “discharge gap' for adult survivors. This model utilizes burn care nurses in advanced roles to coordinate longitudinal care and deliver specialized support such as home-based scar management and psychosocial assistance through telehealth and structured telephone follow-ups (36). By extending professional support into the home environment, this program demonstrates how decentralized, nurse-led interventions can effectively sustain functional recovery in settings where specialized therapists are scarce

### 4.4 Ensure financing for burn rehabilitation in universal health coverage and insurance schemes

To ensure that burn care in regions like Sub-Saharan Africa moves beyond mere survival toward a standard of functional recovery and social dignity, national health authorities must prioritize the integration of rehabilitation into Universal Health Coverage (UHC) frameworks and essential health benefit packages. Ministry of Health policy planners should explicitly include burn rehabilitation, pressure garments, and custom splints in the Essential Health Benefits Package (EHBP). This policy ensures that insurance schemes recognize these services as “function-preserving' and life-saving, protecting survivors from the catastrophic health care costs (5, 29, 30). By mandating insurance coverage for longitudinal care and assistive devices, health systems can prevent avoidable disability and address the significant reintegration burden that currently limits return to work (RTW) rates for survivors with contractures to just 45% (18).

### 4.5 Develop community-based rehabilitation (CBR) arms within burn services

CBR teams should be trained and equipped to support burn survivors post-discharge, particularly in remote or peri-urban settings. CBR should include home-based exercises, scar management, psychosocial support, and caregiver education to reduce risk of contractures and promote reintegration.

### 4.6 Invest in burn rehabilitation workforce development

Establishment of formal BSc and MSc degree programs for Physiotherapists and Occupational Therapists. With only South Africa and Nigeria currently offering advanced degrees, countries like Rwanda are left with a mere 0.02 OTs per 10,000 people (26). Resilience requires targeted scholarships for rural students, mentorship networks linking urban centers to district hospitals, and mandated continuous professional development aligned with ISBI and Interburns standards (15, 37). Investing in these academic pathways is critical for transitioning health systems from informal care to a sustainable, specialized workforce capable of managing the complex trajectory of burn recovery.

### 4.7 Create data systems to track rehabilitation outcomes

Health systems in Sub-Saharan Africa should establish robust data systems within national health information structures to track long-term recovery of burn survivors using feasible, locally relevant rehabilitation indicators (4). While specialized outcome measures such as the Burn Specific Health Scale-Brief (BSHS-B), the Vancouver Scar Scale (VSS), or advanced goniometry are widely used in high-income settings, these tools are often impractical for routine implementation in low-resource environments (1, 4). Instead, registries should prioritize broader, actionable metrics such as the presence and severity of contractures, rates of return to school or work, and frequency of patient contact with rehabilitation professionals (4, 18).

Developing or adapting these locally meaningful instruments allows health systems to assess whether rehabilitation services are effectively reaching survivors within their specific socioeconomic and infrastructural constraints (4). Aligning these metrics with the World Health Organization Global Burn Registry further ensures that data collected informs national policy, guides resource allocation, and strengthens overall service delivery (6, 13, 25, 29, 39). This approach provides a practical foundation for routine monitoring, supports equity in access to rehabilitation, and creates an evidence base for continuous improvement in burn care.

### 4.8 Promote survivor-led peer support and advocacy

To enhance recovery outcomes in resource-limited settings, it is essential to promote survivor-led peer support and advocacy as a low-cost, high-impact intervention. Burn survivors frequently face “unexpected body suffering” and the profound trauma of “losing face,” particularly when injuries affect visible areas like the head and neck (21, 22). These survivor-led peer support and advocacy are vital for enhancing recovery, as trained survivors serve as functional role models who improve patient adherence to often painful rehabilitation protocols through the power of shared lived experience (22). The survivor-led networks also provide a critical psychosocial venue for survivors to navigate the trauma of social mockery and family abandonment, thereby improving self-esteem and facilitating community reintegration. Furthermore, collective advocacy from these groups is essential for demanding service equity and ensuring that longitudinal rehabilitation is prioritized as a fundamental component of the health system (11, 40).

## 5 Conclusion

Burn rehabilitation is an essential yet neglected pillar of recovery in many health systems across Sub-Saharan Africa. The goal is not only to save lives, but to restore function, dignity, and opportunity. Failing to invest in timely rehabilitation perpetuates cycles of disability, poverty, and exclusion especially for children, women, and survivors in rural settings.

Strengthening rehabilitation services will not compete with acute burn care; rather, it will complete it. Simple, context-specific strategies can prevent life-altering contractures and transform “post-burn” recovery. Now is the time to bridge the gap between wound closure and true healing by embedding rehabilitation into every level of burn care from bedside to community, and from policy to practice.

Highlights the overlooked burden of burn-related disability in Sub-Saharan Africa and the consequences of neglected rehabilitation services.

Provides practical, context-specific recommendations to integrate rehabilitation into burn care systems, even in low-resource settings.

Advances the global discourse on disability inclusion by positioning burn rehabilitation as a core component of Universal Health Coverage and the right to health.

Advocates for survivor-led solutions and equity-centered policies that prioritize functional recovery, not just survival, in burn care delivery.

## Data Availability

The original contributions presented in the study are included in the article/Supplementary Material, further inquiries can be directed to the corresponding author.
